# Antioxidant and Antimicrobial Activity of Rosemary, Pomegranate and Olive Extracts in Fish Patties

**DOI:** 10.3390/antiox8040086

**Published:** 2019-04-03

**Authors:** Lorena Martínez, Julián Castillo, Gaspar Ros, Gema Nieto

**Affiliations:** 1 Department of Food Technology, Nutrition and Food Science, Veterinary Faculty University of Murcia, Campus de Espinardo, 30100 Espinardo, Murcia, Spain; lorena.martinez23@um.es (L.M.); gros@um.es (G.R.); 2 Research and Development Department of Nutrafur-Frutarom Group, Camino Viejo de Pliego s/n, 80320 Alcantarilla, Murcia, Spain; j.castillo@Nutrafur.com

**Keywords:** antioxidant, antimicrobial, hydroxytyrosol, rosemary, pomegranate, fish, volatile compounds

## Abstract

Natural extracts (rich in bioactive compounds) that can be obtained from the leaves, peels and seeds, such as the studied extracts of Pomegranate (P), Rosemary (RA, Nutrox OS (NOS) and Nutrox OVS (NOVS)), and olive (*Olea europaea*) extracts rich in hydroxytyrosol (HYT-F from olive fruit and HYT-L from olive leaf) can act as antioxidant and antimicrobial agents in food products to replace synthetic additives. The total phenolic compounds, antioxidant capacity (measured by 2,2-diphenyl-1-picrylhydrazyl (DPPH), 2,2-Azinobis (3-ethylbenzothiazolin) -6-sulphonic acid (ABTS), Ferric Reducing Antioxidant Power (FRAP), and Oxygen Radical Absorbance Capacity (ORAC_H_)) and their antimicrobial power (using the diffusion disk method with the *Escherichia Coli, Lysteria monocytogenes,* and *Staphilococcus Aureus* strains) were measured. The results showed that all the extracts were good antioxidant and antimicrobial compounds in vitro. On the other hand, their antioxidant and antimicrobial capacity was also measured in fish products acting as preservative agents. For that, volatile fatty acid compounds were analysed by GS-MS at day 0 and 11 from elaboration, together with total vial count (TVC), total coliform count (TCC), *E. Coli,* and *L. monocytogenes* content at day 0, 4, 7 and 11 under refrigerated storage. The fish patties suffered rapid lipid oxidation and odour and flavour spoilage associated with slight rancidity. Natural extracts from pomegranate, rosemary, and hydroxytyrosol delayed the lipid oxidation, measured as volatile compounds, and the microbiological spoilage in fish patties. Addition of natural extracts to fish products contributed to extend the shelf life of fish under retail display conditions.

## 1. Introduction

The food industry generates an enormous amount of waste in form of skins, seeds and leaves, whose disposal is a problem for the environment and expensive for companies concerned. Many residues from fruits, which are rich source of phenolic compounds, can be extracted and used by food industries as antioxidant and antimicrobial preservatives.

In this work, the antimicrobial and antioxidant capacity of several extracts from rosemary, pomegranate, and hydroxytyrosol were measured. These extracts were chosen because of their beneficial effects for human health and their notable antioxidant properties. *Rosmarinus officinalis* L. is a natural woody perennial green herb from the Mediterranean region rich in vitamins A, C, B1, B6 and B9, minerals such as Mg, Ca, Cu, Fe and Mn, as well as phenolic compounds (diterpenes and rosmarinic acid). The regular consumption of this herb has been seen to have many beneficial effects for human health [1,2] acting as a powerful antioxidant and antibacterial agent [3]. Furthermore, pomegranate extract, from *Punica granatum* fruit, contains large quantities of phenolic compounds (ellagitannins, flavonoids, punicalagin, ellagic acid, vitamin C and minerals). For this reason, pomegranate has been used in medical applications for more than 2000 years. Among its beneficial effects for the human body [4,5], pomegranate peel extracts and other subproducts obtained from this fruit, such as juice or seeds, have shown high antioxidant and antimicrobial capacities, with a great scavenging power, preventing microbiological growth of different bacteria [6,7]. On the other hand, oleuropein is the main phenolic compound in olive (*Olea europaea*) tree and the precursor of hydroxytyrosol, which is also extracted from leaves and vegetation waters from the manufacture of olive oil. This phenolic phytochemical is considered the most antioxidant compound after gallic acid, whose consumption has many beneficial effects for the skin, eyes and immune system, and acting as an antimicrobial, anti-infammatory and anticancer agent [8,9,10].

As these extracts act as antioxidants, antimicrobials and hence preservatives of shelf life food products, it is interesting to investigate about their action in a food matrix. In this case, fish has been chosen due to it is an important source or omega-3, particularly eicosapentaenoic acid (EPA), and docosahexaenoic acid (DHA), essential fatty acids for the protection against autoimmune, inflammatory and cardiovascular diseases [11]. However, fish consumption has decreased in populations groups under 14 years old, thus manufacturation of fish products as “burgers” could be a good option to stimulate fish consumption among young people [12]. In the same way, synthetic additives are widely used to avoid the microbiological, enzymatic and oxidative degradation of these kinds of products. As substitution of synthetic additives by natural extracts is gaining in importance in the food industry [13], the use of natural extracts as preservatives in fish products could be an excellent way to produce a saludable product with high nutritional level, Clean label, and without synthetic additives. Alternatively, pomegranate has already demonstrated its antioxidant capacity after addition to chub mackerel minced muscle during frozen storage [14]. In this case, 2% pomegranate seed extract inhibited the formation of substances related with lipid oxidation regard to the control sample. Similarly, rosemary oil at 0.2%, 1% and 3% added to minced rainbow trout had a positive effect on the freshness indicators, oxidative stability, fatty acid and biogenic amine contents during refrigerated storage [15]. In addition, hydroxytyrosol has also showed an important inhibition of the formation of lipid oxidation products in foodstuffs rich in fish lipids (bulk cod liver oil, cod liver oil-in water emulsions and frozen minced horse mackerel) [16], while in a general view, hydroxytyrosol has also demonstrated to have excellent antioxidant properties in a nanostructured starch developed as active food packaging [17].

Briefly, the objective of this work was to make a comparative study of pomegranate, rosemary and hydroxytyrosol extracts measuring their antioxidant and antimicrobial capacities, as in vitro, following several methods, as antioxidants and antimicrobials in a food matrix: fish patties.

## 2. Materials and Methods

### 2.1. Plant Extracts

The plant extracts used were: Pomegranate (P), Rosemary: Rosmarinic acid extract (RA), Nutrox OS (NOS), Nutrox OVS (NOVS), Hydroxytyrosol from fruit (HYT-F), and from leaf (HYT-L). All extracts were supplied by Nutrafur-Frutarom Group (Alcantarilla, Murcia, Spain) and obtained from the corresponding dry vegetal materials using a maceration process including, as an initial step, solid-liquid extraction with different ethanol-water mixtures. Pomegranate extract (P) was obtained from dehydrated lignocellulosic materials of fruits by means of a hydro-alcoholic extraction, filtration of the vegetal material, evaporation of the ethanol and crystallisation in aqueous medium, followed by concentration and drying. De-oiled rosemary leaf was used as raw material to obtain the rosemary extracts used in this study. The water-soluble rosemary extract (RA) was obtained by extraction in aqueous medium and simple filtration of the plant material, concentration and drying. Rosemary extracts not soluble in water were obtained by extraction with acetone-water, filtration of the plant material, concentration and drying. Subsequently, the solid obtained was dissolved in different excipients to obtain the two extracts used in this study, sunflower oil (NOS) and sunflower oil plus lecithin (NOVS). HYT-L was made by extraction in ethanol-water medium, filtration of the plant material, evaporation of the solvent and subsequent thermal treatment. Finally, the water insoluble materials were filtered and dried. While HYT-F was obtained by extraction from the dry plant material with ethanol (vegetation waters), decantation, evaporation of the solvent, drying, recrystallisation of aqueous medium, elimination of insoluble materials in water and drying. All the drying processes were carried out in a vacuum and at a reduced temperature (50–70 °C).

#### 2.1.1. Determination of Extract Composition (HPLC)

For the quantification of phenolics in the P extract, it was dissolved in dimethylsulfoxide (DMSO) in the ratio of 4 mg/mL; this solution was filtered through a 0.45 µm nylon membrane. The HPLC equipment used for all the extracts was a Hewlett-Packard Series HP 1100 equipped with a diode array detector. The stationary phase was a C_18_ LiChrospher 100 analytical column (250 × 4 mm i.d.) with a particle size of 5 µm (Merck, Darmstadt, Germany) thermostated at 30 °C. The flow rate was 1 mL/min and the absorbance changes were monitored at 280 nm. The mobile phases for chromatographic analysis were: (A) acetic acid/water (0.1:99.5) and (B) methanol. An initial isocratic period for 15 min with 100% (A) was run, after, a linear gradient was run from 100% (A) to 90% (A) and 10% (B) for 15 min (30 min total time), and it was maintained for 10 min (40 min total time), before reequilibrating in 10 min (50 min, total time) to initial composition. Punicalagin in P was identified and quantified by comparation of their retention times with the corresponding standard and by their UV spectra obtained with the diode array detector.

For the quantification of diterpenes in rosemary extracts (mainly carnosic acid and carnosol), for NOS and NOVS extracts, each extract was dissolved in methanol in variable ratios between 0.2 and 4 mg/mL, depending on the diterpene concentration expected for each extract; the solution was filtered through a 0.45 µm nylon membrane. The flow rate was 0.75 mL/min, and the elution was monitored at 230 nm. HPLC was used for the separation of the different diterpenes present in the rosemary extracts. The mobile phase for chromatographic analysis was an isocratic single step of acetonitrile (65%), water (35%) and phosphoric acid (0.2%) for 25 min. Diterpenes were quantified by comparing the chromatographic areas of the corresponding standards. On the other hand, for the quantification of rosmarinic acid in rosemary extracts (RA), the solid was dissolved in water at 5 mg/mL for analytical chromatography; this solution was filtered through a 0.45 µm nylon membrane. The flow rate was 1 mL/min. The absorbance changes were monitored at 340 nm.

For the quantification of phenolics in the olive (*Olea europaea*) extracts (HYT-F and HYT-L), the extract was dissolved in dimethylsulfoxide (DMSO) in the ratio of 5 mg/mL; this solution was filtered through a 0.45 µm nylon membrane. The flow rate was 1 mL/min and the absorbance changes were monitored at 280 nm. The mobile phases for chromatographic analysis were: (A) acetic acid/water (2.5:97.5) and (B) acetonitrile. A linear gradient was run from 95% (A) and 5% (B) to 75% (A) and 25% (B) for 20 min; it was then changed to 50% (A) and (B) in 20 min (40 min, total time); in 10 min it was changed to 20% (A) and 80% (B) (50 min, total time), before reequilibrating in 10 min (60 min, total time) to initial composition. Phenolic compounds in olive extracts were identified and quantified by comparation of their retention times with the corresponding standard and by their UV spectra obtained with the diode array detector [18].

Pure standards for HPLC quantification: hydroxytorosol (Code 4999S, Extrasynthése, Genay, France); carnosic acid (Sigma, Code C-0609, Madrid, Spain); carnosol (Sigma, Code C-9617, Darmstadt, Germany); rosmarinic acid (Extrasynthése, Code 4957S, Genay, France); punicalagin (Sigma, Code P-0023, Darmstadt, Germany).

#### 2.1.2. Total phenolic content (TPC)

The total phenolic content (TPC) was determined quantitatively using the Folin–Ciocalteu reagent and gallic acid as the standard [19,20]. Each extract was diluted with water or ethanol, according to its polarity, in a 1000 ppm solution. Then, 2 mL of 2% Na_2_CO_3_ was added to 100 µL of sample and a standard solution of gallic acid. After, 100 µL of 10:1 Folin–Ciocalteau phenol reagent was added. Following incubation for 30 min, the absorbance was measured at 750 nm. The TPC was expressed as mg gallic acid equivalents (GAE) per g extract.

#### 2.1.3. Antioxidant Activity

The antioxidant activity was also measured using the 2,2-diphenyl-1-picrylhydrazyl (DPPH) free radical scavening method, described by Brand-Williams et al. [21] and Sánchez-Moreno et al. [22]. Each extract was diluted with water or ethanol in a 1000 ppm solution. A DPPH radical solution was prepared with 0.0063 g DPPH in 250 mL ethanol. Then, 3.9 mL of DPPH radical solution was added to 100 µL of sample and a standard solution of 500 µM Trolox. After incubation in the dark at room temperature for 30 min, the absorbance was measured at 515 nm. The % chelating activity was calculated using the following formula: [(Abs DPPH − Abs Sample)/Abs DPPH] × 100 [23].

Radical cation scavenging capacity against ABTS+ radical (2,2-Azinobis (3-ethylbenzothiazolin) -6-sulphonic acid) was measured as by Re et al. [24]. Each extract was diluted with water or ethanol in a 1000 ppm solution. ABTS radical cations were prepared by reacting 7 mM ABTS (2,2-Azinobis (3-ethylbenzothiazolin) -6-sulphonic acid) with 2.45 mM potassium persulphate (1:1, *v*/*v*) pH = 7.4. This solution was diluted with distilled water to an absorbance of 0.7000 at 734 nm. Then, 1 mL of ABTS solution was added to 100 µL of sample and standard solution of 500 µM Trolox. After incubation for 2 min, the absorbance was measured at 734 nm. The % chelating activity was calculated using this formula: [(Abs ABTS − Abs Sample)/Abs ABTS] × 100.

The hydrophilic antoxidant capacity was measured using the ORAC (Oxygen Radical Absorbance Capacity) method described by Prior et al. [25]. The reaction was carried out in a phosphate buffer (0.075 M, pH 7.0). For this, 20 µL of sample, at different concentrations, and Trolox standard solutions (6.25, 12.5, 25, 50 µM), were pipetted into the wells of a 96-well black microplate. When the microplate was ready, 200 µL of 0.04 µM fluorescein were dispensed into each well. Samples were incubated for 15 min at 37 °C in the dark and the reaction was started by adding 20 µL of 40 mM AAPH (2,2′-Azobis(2-amidinopropane)dihydrochloride) to each well. The flourescence decay was measured every 2 min at 37 °C using the microplate reader Biotek Synergy HT at 485 nm excitation and 538 nm emission until zero fluorescence was detected. All samples were prepared in triplicate and at a minimum of three different concentrations. The antioxidant activity of the sample was expressed as µM of Trolox Equivalents (TE) per g of extract, with the following formula: *(C* × *DF)/a;* where *C* is obtained from the area under the fluorescence decay curve of fluorescein in the presence of sample (AUC net = AUC sample − AUC blank); *DF* is the dilution factor; and *a* is the weight of the sample. A Biotek Synergy HT fluorescent microplate reader (Biotek Instruments, Winooski, VT, USA) was used with an excitation wavelength of 485 nm and an emission wavelength of 528 nm; and a UV2 spectrophotometer (Pye Unicam Ltd., Cambridge, UK) at different wavelengths depending on the method to be performed.

The total antioxidant activity was also determined using the method described by Benzie and Strain [26] with some modifications. Each extract was diluted with water or ethanol in a 1000 ppm solution. Then, the FRAP reagent was prepared with 20 mL 300 mmol/L acetate buffer pH = 3.6, 2 mL 20 mmol/L FeCl_3_·6H_2_O and 2 mL 10 mmol/L TPTZ (2,4,6-tripyridyl-s-triazine) in 40 mmol/L HCl. Then, 1 mL of the FRAP reagent was added to 100 µL of sample and a standard solution of 500 µM Trolox. After incubation for 4 min, the absorbance was measured at 593 nm. The antioxidant power was expressed as µM Trolox Equivalents (TE) per g extract.

#### 2.1.4. Antimicrobial Activity

All extracts were tested against Gram-negative bacteria: *Escherichia Coli* O157:H7 ATCC 25922 CECT 434*;* and Gram-positive bacteria: *Lysteria Monocytogenes* KCTC 3569 CECT 7467 and *Staphilococcus Aureus* ATCC 25923 CECT 435. The antimicrobial activity of the different extracts was measured following the diffusion disk method described by Chanwitheesuk et al. [27] with some modifications, using a bacterial cell suspension with an equilibrated concentration to a 0.5 McFarland standard or 10^5^–10^6^ cfu/mL. Each bacterial suspension was spread on a Mueller-Hinton agar plate. Sterile paper discs of 6 mm diameter were impregnated with 30, 60 and 90 µL of each test sample. Chloramphenicol (30 µg), a standard antibiotic was used as a positive control, while discs with the solvents used for extraction were used as negative controls, water or MeOH. The plates were incubated at 37 °C for 24 h. After incubation, the inhibition zones appearing around the discs were measured and recorded. The values, expressed in mm, were averages of six measurements per disc, taken at six different points in order to minimise errors.

### 2.2. Fish Patties

Antioxidant and antimicrobial extracts were also tested in fish patties that incorporated them in each sample formula.

#### 2.2.1. Samples Elaboration

Ultrafrozen skinless hake fillets (*Merluccius capensis, Merluccius paradoxus*) (Pescanova España S.L.U.) were bought in a local supermarket and thawed in refrigeration for 24 h before making into fish patties. Fish was minced in an electric mincer (Bosch, Germany) for 2 min, mixing all the ingredients of each one of seven samples, represented in Table 1. Afterwards, fish patties were formed (50 g) and packed in aerobic conditions. Samples were stored at 4 °C until analysis, at day 0, 4, 7 and 11. 20 fish patties were formed for each batch and analysis were carried out by triplicated.

#### 2.2.2. Volatile Organic Compounds Analysis

Lipid oxidation was related with the concentration of volatile organic compounds (VOCs). For that, 5 g of fish burger samples were placed in glass vials. Volatile compounds were extracted using solid-phase microextraction (SPME). A SPME device (Supelco, Bellefonte, PA, USA) containing a fused-silica fibre (10 mm length) was used. Extraction was performed at 35 °C for 30 min. Once sampling was finished, the fibre was withdrawn into the needle and transferred to the injection port of the gas chromatograph-mass spectrometer (GC-MS) system. Volatiles were analysed in duplicate in all samples at days 0 and 11 from elaboration. Analyses were performed on a Hewlett-Packard 6890 N Series GC gas chromatograph fitted with a HP 5973 mass spectrometer and an MSD Chemstation (Hewlett-Packard, Palo Alto, CA, USA). A split injection port was used to thermally desorb the volatiles from the SPME fibre onto the front of the DB-624 capillary column (J&W scientific: 30 m × 0.25 mm id, 1.4 µm film thickness). Helium was used as a carrier gas with a linear velocity of 36 cm/s. The temperature programme was: 40 °C for 2 min and then raised to 100 °C at 3 °C/min; then from 100 to 180 °C at 5 °C/min; total run time 50.8 min. The mass spectra were obtained using a mass selective detector working in electronic impact at 70 eV, with a multiplier voltage of 1953 V and collecting data at a rate of 6.34 scans/s over the range *m*/*z* 40–300. Compounds were identified by comparing their mass spectra with those contained in the NIST05 (National Institute of Standards and Technology, Gaithersburg) library.

#### 2.2.3. Microbiological Analysis

Microbiological growth of total vial count (TVC), total coliform count (TCC), *E. Coli, S. Aureus,* and *L. monocytogenes* was determined on days 0, 4, 7 and 11 from elaboration. Mass seeding was performed on Rapid E. Coli (to determine *E. Coli* and TCC), PCA (TVC), and Rapid L. mono (*L. monocytogenes*). All media were sterilised at 121 °C for 20 min according to product indications. Peptone water (OXOID, Ltd. CM0087 Basingstoke, Hampshire, United Kingdom) was used to make the dilutions. A laminar flow hood (Telstar, BIO-II-A, Spain) was used to carry out the analysis. Finally, plates were incubated for 24 h at 45 °C for *E. Coli*, 24 h at 37 °C for TCC, 48 h at 37 °C for TVC, and 48 h at 37 °C for *L. monocytogenes*. Results were obtained by triplicated and expressed in cfu/g.

### 2.3. Statistic Analysis

Data were analysed with the statistical package SPSS 15.0 (Statistical Package for the Social Science for Window (IBM, Armonk, New York, USA). The antioxidant and antimicrobial capacity were analysed using ANOVA. A value of *p* < 0.05 was considered statistically significant. Pearson’s correlation was applied out to test differences between groups.

## 3. Results

### 3.1. Characterisation of Natural Extracts (HPLC)

**Figure 1:**
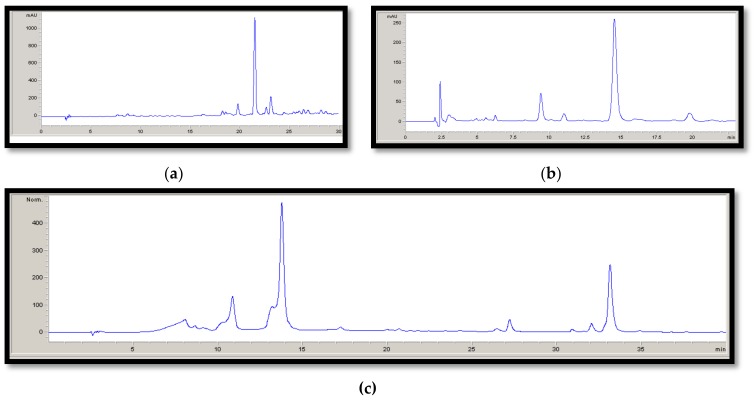

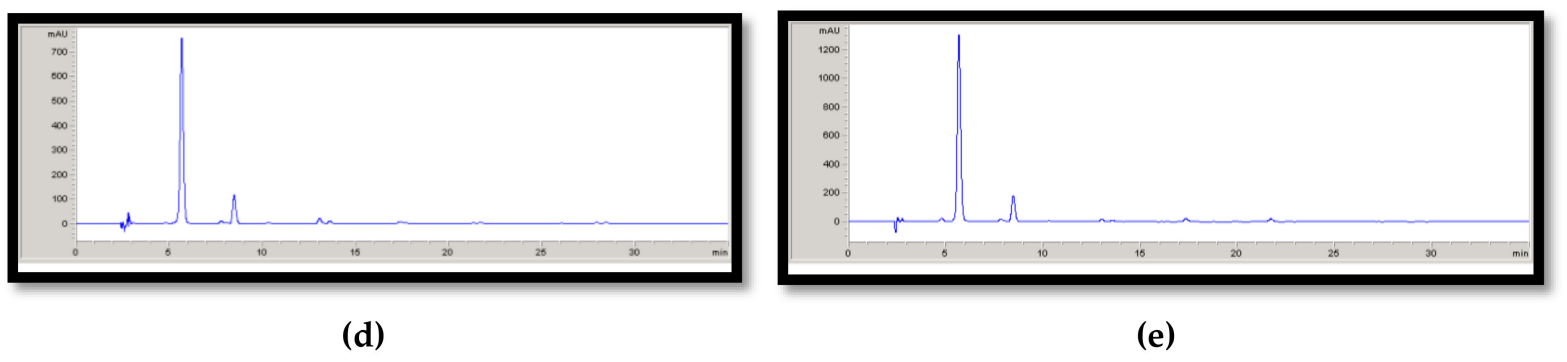
HPLC chromatograms for natural extract. (**a**) RA: Rosemary extract rich in Rosmarinic Acid, (**b**) NOS: Rosemary extract rich in diterpenes and NOVS: Rosemary extract rich in diterpenes and with lecitin as emulsifier, (**c**) P: Pomegranate extract, (**d**) HYT-F: Hydroxytyrosol extract obtained from olive fruit, (**e**) HYT-L: Hydroxytyrosol extract obtained from olive leaf.

The antioxidant and antimicrobial capacities of these extracts depend on the concentration of the phenolic compounds. Extracts obtained from *Rosmarinus officinalis* L. contained 8.10% of rosmarinic acid (RA), and 5.76% of diterpenes (NOS and NOVS), more specifically, carnosic acid and carnosol. P had 41.38% of total punicalagins, as principal bioactive compounds. Otherwise, hydroxytyrosol extracts, obtained from different parts of the olive tree (*Olea europaea*) during the manufacture of olive oil, had different concentrations of the bioactive compound: HYT-F had 11.25% hydroxytyrosol, while HYT-L presented 7.26%. HPLC chromatograms for each extract are presented in Figure 1.

#### 3.1.1. Total Phenolic Content (TPC)

Determination of the total phenolic content by means of the Folin–Ciocalteau method allows a comparative evaluation of the content of this kind of compounds, considering, at molecular level, the significant structural difference between the various polyphenols present in the extracts being analysed. The results obtained (mg GAE/g) are shown in Table 2.

HYT-F showed the highest quantity of phenolic compounds, with 41.44 mg GAE/g, followed by P, NOS, RA, HYT-L, and NOVS. This last one with 35.95 mg GAE/g, 13.2% less than the first extract. All the extracts showed more than 35 mg GAE/g. In that way, hydroxytyrosol (HYT-F and HYT-L), rosmarinic (RA, NOS and NOVS), and pomegranate extracts had similar total phenolic amounts, between 36 and 41 mg GAE/g.

#### 3.1.2. Antioxidant Activity

The chelating activity percentages obtained using two different methods, are also shown in Table 2. ABTS + radical cation assay and the DPPH free radical scavenging method were used. The capacity of P, HYT-L and RA to scavenge the ABTS + radical was higher than 80%, while the chelating activity of HYT-F, NOVS, and NOS was of 79.62%, 70.61% and 70.17%, respectively. On the other hand, scavenging ability of P was also the highest by measuring the stability of the DPPH radical, 92.55%. The chelating activity of HYT-L, NOVS, NOS, RA, and HYT-F ranged from 89% to 78%. Thus, the extracts with greatest chelating capacity were those obtained from pomegranate, hydroxytyrosol and rosemary, which is related with their high content of phenols, such as, punicalagin, hydroxytyrosol and rosmarinic compounds (carnosic acid, carnosol, and rosmarinic acid), respectively.

The hydrophilic antioxidant capacity of the natural extracts obtained by measuring the oxygen radical absorbance is shown in Table 2. However, in this case, the extract with the greatest antioxidant activity was HYT-L (147.46 µM TE/g), followed by P (146.39 µM TE/g), HYT-F (140.54 µM TE/g), RA (123.2 µM TE/g), NOVS (49.16 µM TE/g) and NOS (45.18 µM TE/g). As it can be appreciated, a great significant difference (*p* < 0.05) exists among rosmarinic extracts (NOS and NOVS) rich in diterpenes (carnosic acid and carnosol) and RA rich in rosmarinic acid, together with P, HYT-L and HYT-F. This fact could be explained by lipophilic activity of NOS and NOVS, because of this measurement is carried out in a hydrophilic system. In this way, the rest of extracts are watersoluble, as P, as well as HYT-L, HYT-F and RA present dual affinities, both to polar and non-polar solvents.

The efficiency of the natural plant extracts to reduce Fe^+++^ to Fe^++^ as a antioxidant power measured is also presented in Table 2 expressed in µM Trolox Equivalents (TE)/g. Obtained data showed as that all the extracts have a good and similar ferric reducing antioxidant power, ranging from 73.8 µM TE/g (RA) to 61.3 µM TE/g (P). The order of antioxidant activity using this method was RA > NOVS > HYT-F > HYT-L > NOS > P. All the extracts had high levels of reducing power, which indicated the presence of some compounds that are electron donors and could react with free radicals to convert them into more stable products.

#### 3.1.3. Antimicrobial Activity

Data obtained from measuring the antimicrobial capacity of different extracts are presented in Table 3. The results differed according to the bacterial strain used, *L. monocytogenes* KCTC 3569 CECT 7467 (Gram-positive)*, S. Aureus* ATCC 25923 CECT 435 (Gram-positive) or *E. Coli* O157:H7 ATCC 25922 CECT 434 (Gram-negative). All the extracts showed a lower growth inhibition capacity than cloramphenicol (positive control), a broad spectrum antibiotic.

HYT-L, followed by HYT-F had the highest antimicrobial capacity values against *S. Aureus* ATCC 25923 CECT 435 (gram-positive) growth, because of they prestented 28.1 and 25.2 mm of growth inhibition, respectively. These extracts were followed by RA, P, NOS and NOVS. In this case, clearly, the most active compound was the hydroxytyrosol, as obtained from olive leaves as from olive fruits. In second place was RA, which is the most similar in terms of its molecular structure. Both of them provide an opportunity to study the action mechanism of these compounds in future research. However, the inhibitory capacity of the rest of the compounds was lower and does not allow any structure-activity hypothesis to be proposed.

On the other hand, the gram-positive bacterium *L. monocytogenes* KCTC 3569 CECT 7467 is the most resistant strain to phenolic compounds. In this case, P was the most antimicrobial with 15.3 mm growth inhibition, which corresponds with 44.1% of the positive control, chloramphenicol. This was followed by HYT-L, NOVS, NOS, HYT-F and RA. It can be considered, then, that all the studied compounds showed similar inhibitory activities against this microorganism.

Finally, NOS, was the most antimicrobial extract against the gram-negative bacteria *E. Coli* O157:H7 ATCC 25922 CECT 434, with 20 mm of growth inhibition, 40.6% less than chloramphenicol. This phenolic extact was followed by NOVS, RA, P, HYT-L and HYT-F. In this case, of great interest and significance is the fact that fat-soluble compounds such as terpenoids had a higher inhibitory capacity against the growth of gram-negative bacteria. This was followed by the rest of extracts, all of which showed similar values of antimicrobial activity, making it difficult to offer any considerations on their structure-activity.

### 3.2. Influence of Natural Extracts in Oxidative and Microbiological Damage in Fish Products

#### 3.2.1. Volatile Organic Compounds

Table 4 shows the results obtained from the of GS-MS analysis of the volatile organic compounds: 1-Penten-3-ol, hexanal, 2-nonanone, 1,6-octadien-3-ol, octanal, pentadecane in fish patties. In general, all the volatile compounds analysed increased (*p* < 0.05) from the beginning of storage for all the treatments. These results point to the degradation that is shown in fish patties, that is due to oxidation phenomena, as most straight chain aldehydes are derived from the oxidation of unsaturated fatty acids.

Hexanal and 1,6-octadien-3-ol were the dominant aldehyde in the fish patties meat in all the groups. Hexanal values ranged from 0.1 mg/kg, in day 0, to 5.14 mg/kg after 11 days, in control samples. These values are in the same line than those reported by Brunton et al. [28], who found hexanal values of 4.01 l/g in cooked turkey stored for 6 days at 4 °C.

Differences in the mean hexanal levels between C and patties with natural extracts were significant (*p* < 0.05) on day 11. On day 11, NOVS showed lower (39%) hexanal values than C, meaning that Rosemary extracts improved lipid stability of the fish patties. In this sense, Shahidi, Yun, and Rubin [29] reported that such an increase in hexanal is a good indicator of lipid oxidation. Indeed, these authors suggested hexanal as a valid indicator of oxidative stability and flavour acceptability in cooked ground meat.

The behaviour of nonanal and 1-penten-3-ol was similar to hexanal, both increasing (*p* < 0.05) during storage and showing significant differences between C and natural extracts samples on day 11. Nonanal is a waxy flavor and descriptors, while 1-penten-3-ol is amongst the compounds responsible for the rancid odour in mayonnaise. Note the absence of significant differences in 2-nonanone and pentadecane on day 11.

#### 3.2.2. Microbiological Content

Antimicrobial capacity of different extract was also proved in fish products elaborated from thawed hake. Consequently, microbiological results of fish patties at days 0, 4, 7 and 11 from elaboration, are shown in Table 5.

As it can be appreciated, TVC results after 11 days of refrigerated storage were lower in samples that incorporated HYT-F and HYT-L in their formula, followed by P, control sample, NOVS, NOS and RA. While, TCC results showed as both pomegranate and rosemary extracs, diterpens rich extracts (NOS and NOVS) and rosmarinic acid rich extract (RA) obtained the lowest results in comparison with the control sample, or samples that incorporated HYT to their formulas. These last results can be related with antimicrobial activity of all the extracts against *E. Coli* O157:H7ATCC 25922 CECT 434. On the other hand, results obtained from analysis of *E. Coli* and *L. monocytogenes* did not present significant differences (*p* < 0.05) among incorporation of natural extracts. In addition, it must be taken into account that only samples enriched with HYT, both from fruit or leaf, did not exceed the limit stablished by European legislation regarding to TVC in fish products (5,000,000 cfu/g). In parallell, only P and rosemary extracts (RA, NOS, and NOVS) kept fish product samples below legal microbiological safety limits of TCC (50,000 cfu/g). However, all the natural extracts, including the control sample prevented against microbiological growth of *E. Coli* and *L. monocytogenes.*

## 4. Discussion

Firstly, the obtained results of total phenolic content agrees with previous findings by other authors using the Folin-Ciocalteau method or by HPLC [30,31,32]. Results obtained in the present spectrophotometric determination were not strictly correlated with the data obtained by HPLC analysis, which is due to the different response factors of each of the polyphenol structures present in the extracts (punicalagins, rosmarinic acid, carnosic acid, carnosol and hydroxytyrosol) regarding the pattern used, as the gallic acid in this case. It is difficult to make a structural interpretation of the results obtained for the antioxidant capacity measurements using the studied methods, although, clearly, some factors are related with the molecular structure of the active substances: the presence of phenols, their conjugation and polymerisation, cathecol and/or gallate groups presence, etc. In both methods, P shows the best results, probably, due to the presence of some conjugated polyphenol structures and a significant amount of gallic acid groups (tri-hydroxy phenol structures). Regarding the olive extracts, HYT-L, with its lower level of hydroxytyrosol than HYT-F, as principal active compound, showed a higher antioxidant capacity in both models, making it one of the most powerful extracts. This fact could originate from the presence of flavonoid compounds in combination with the hydroxytyrosol, providing a synergistic effect in terms of antioxidant activity. RA is the most structurally similar extract to olive extracts, due to the presence of rosmarinic acid as an active compound. This substance could be termed “double-hydroxytyrosol,” only for their structural similarity, perhaps for this reason both extracts showed proximate chelating activity values. The difference among rosemary extracts was significant. The water-soluble extract (RA) was more active in the ABTS method, while liposoluble extracts (NOS, and NOVS) showed a higher activity in the DPPH model. This behaviour could be explained by the different chemical structure of the radical used in each technique and by the different properties of the molecular structures of phenylpropanoids (rosmarinic acid) and diterpens (carnosic acid and carnosol). However, both structures have a cathecol group and a carborxylic acid group. These results can be compared with previous research. For example, Hmid et al. [33] and Elfalleh et al. [34] obtained similar values for pomegranate extracts using the same methods, as well as hydroxytyrosol [35] and rosemary extracts [36,37].

Hydrophilic ORAC is one of the most widely used methods for evaluating antioxidant capacity, but it is clear that the results may be conditioned not only by the antioxidant capacity of each compound, but also by the physical and chemical properties, particularly its water solubility. Pomegranate and olive extracts obtained similar values for their antioxidant activity unrelated to their origin (leaves, fruit or vegetation water). In this case, the different of cathecol and gallate groups did not seem to be significant. Despite this, HYT-L again showed a higher activity. The antioxidant capacity of RA was lower than that of the above (–15%), although it followed the same order. It seems obvious that the structural similarity goes on establishing a parallellism in the antioxidant activity, also in this model. If not, the lower ORAC activity of the fat-soluble rosemary extracts (diterpens) compared with the hydrosoluble extracts that were already described. Previous researchers, such as Azaizeh et al. [38], obtained similar results analysing hydroxytyrosol in olive (*Olea europaea*) vegetation waters, while Sueishi et al. [39] obtained results that were 50% higher when measuring the seasonal variations of oxygen radical scavenging ability in rosemary leaf extract using the same method. In research carried out by Durante et al. [40], the authors measured the antioxidant activity of diferent extracts from tomato, grape and pomegranate seeds, obtaining similar results as the last. In the same way, previous research obtained similar results to that obtained results by the FRAP method regarding to rosemary [36], pomegranate [33] and hydroxytyrosol [35,41].

Regarding antimicrobial activity, the terpenoid structure did not provide good results, although this does not mean that this compound has a lower antioxidant capacity. While not significant, it is interesting to point out that NOVS, which contains lecitin as an emulsionant, shows higher antioxidant activity than NOS, which does not contain this excipient. This method has been used in much research to test the antimicrobial capacity of many drugs and natural extracts. For example, Laincer et al. [39] measured the antimicrobial activity of several olive phenolics, including HYT, against *E. Coli* and *S. Aureus,* obtaining similar results. Weckesser et al. [42] analysed the antimicrobial activity of plant extracts, such as *Rosmarinus officinalis* L. against bacteria of dermatological relevance, among them *E. Coli* and *S. Aureus,* obtaining similar results using the diffusion disk method. Regarding the P extract, Kharchoufi et al. [7] obtained similar results for pomegranate peel extracts against *Pseudomonas putida, Penicillium digitatum* and *Saccharomyces cerevisae,* but not against any strains used in the present study. Finally, applying rosemary extracts, Santomauro et al. [43] obtained similar results (more than 10 mm of inhibition) in different strains.

Regarding the oxidative and antimicrobial damage of fish products under refrigerated storage for 11 days, all the natural extracts showed an antioxidative effect against formation of volatile compounds related to lipid oxidation.

Table 4 shows that all the volatile compounds analysed are the main components that contribute the most to the emergence of unpleasant notes of flavour, due to the low flavour threshold [44]. In general, the presence of natural extract (especially NOVS) in the fish patties delayed the formation of all the volatile lipid-derived compounds. In the same line, Nieto et al. [45,46] reported lower hexanal values, rancid odour and rancid flavour scores in lamb meat from ewes fed thyme leaves or rosemary by-products, respectively.

As it can be appreciated in Table 5, natural extracts also acted as antimicrobial agents against TVC and TCC proliferation. This behaviour has been previously observed by other researchers using other natural extracts. For example, Del Nobile et al. [47] studied the combined effect of different gas mix compositions (MAP 30:40:30 O_2_:CO_2_:N_2_, 50:50 O_2_:CO_2_, and 5:95 O_2_:CO_2_) and three essential oils (thymol, lemon and grapefruit seed extracts) on fresh blue fish burgers. Results obtained showed as the combination of 110 ppm of thymol, 100 ppm of grapefruit seed extract, or 120 ppm of lemon extract with MAP 5:95 O_2_:CO_2_ was able to maintain the microbial quality of fish burgers for 28 days under refrigerated storage. In the same way, the combined effect of antimicrobial mixtures of chitosan, nisin and sodium lactate with MAP 55:45 CO_2_:N_2_ was able to guarantee the microbial acceptance of hake burgers for 30 days of refrigerated storage [48]. On the other hand, Smaldone et al. [49] have observed that only MAP 5:60:35 O_2_:CO_2_:N_2_ application can extend the microbiological shelf-life of hake burgers for 15 days after elaboration. However, in the present study, modified atmosphere package treatment was not assessed, neither in previous research on fish products using natural extracts from pomegranate, rosemary or olive tree (*Olea europaea*). Likewise, with obtained results it can be concluded that bioactive compounds from studied extracts (P, RA, NOS, NOVS, HYT-L and HYT-F) act as antimicrobial agents, which has also demonstrated in vitro and it is due to their high concentration of phenolic compounds (punicalagin, carnosic acid, carnosol, rosmarinic acid and hydroxytyrosol) with known antimicrobial activity, as it has been exposed in the introduction of this work. For this reason, it is not surprising that their application avoided *L. monocytogenes* or *E. Coli* growth. Nevertheless, is important to know that samples that incorporated rosemary extracts presented TVC growth higher than the control sample at day 11, similar to hydroxytyrosol extracts that showed higher TCC growth than the control. This fact can be explained by the great amount of antioxidant compounds in combination with spices and spice extracts that the commercial mix contained, and which can produce a synergism between them, increasing the shelf-life of fish products.

## 5. Conclusions

It is necessary to use several methods to know the antioxidant capacity of the studied extracts since they are rich in phenolic compounds with different molecular structures and react in different ways when reducting Fe^+++^ or acting against hydroxyl, ABTS and DPPH radicals. Consequently, natural extracts obtained from olive (*Olea europaea*), pomegranate (*Punica granatum*) and rosemary (*Rosmarinus officinalis* L.) are excellent antioxidant agents. The fish patties made with natural extracts showed lower volatile compounds related with lipid oxidation throughout the 11 days of storage under retail display conditions. On the other hand, the most antimicrobial compounds were P against *L. monocytogenes*, HYT against *S. Aureus,* and NOS against *E. Coli*. After applying these extracts in fish products, all of them acted as preservatives, extending the shelf-life for 11 days with a lower microbiological growth in regard to the control sample. Then, it can be concluded that all the extracts are good antioxidant and antimicrobial agents and could be applied in the food industry to extend shelf-life of Clean label fish products.

## Figures and Tables

**Table 1:** Fish patties formulation.

Ingredients	Control	P	RA	NOS	NOSV	HYT-L	HYT-F
Hake (g)	852	875	875	875	875	875	875
Water (ml)	100	100	100	100	100	100	100
Commercial mix (g/kg)	48						
Fibers (g/kg)		25	25	25	25	25	25
Natural extracts (ppm)		200	200	200	200	200	200

Commercial mix^®^: supplied by Catalina Food Solutions, S.L. (El Palmar, Murcia, Spain) and composed by: vegetables fibers, salt, potato starch, stabiliser (Pocessed euchema seaweed (PES) E-407-a), acidity correctors (sodium citrate E-331, and sodium acetate E-262), spices, spice extracts and antioxidant (sodium ascorbate E-301). P: Pomegranate extract, RA: Rosemary extract rich in Rosmarinic Acid; NOS: Rosemary extract rich in diterpenes; NOVS: Rosemary extract rich in diterpenes and with lecitin as emulsifier; HYT-L: Hydroxytyrosol extract obtained from olive leaf; HYT-F: Hydroxytyrosol extract obtained from olive fruit.

**Table 2:** Total phenolic content (TPC) of natural extracts (mg GAE/g) and their antioxidant activity by measuring their ABTS, and DPPH radical scavenging activity, together to their ORAC_HP_, and FRAP (µM TE/g).

Samples	TPC	ABTS	DPPH	ORAC_HP_	FRAP
	mg GAE/g ± SD	% Chelating Activity	% Chelating Activity	µM TE/g ± SD	µM TE/g ± SD
RA	36.37 ± 0.007 ^b^	81.1 ^a^	81.29 ^b^	123.2 ± 0.24 ^b^	73.81 ± 1.52 ^a^
NOS	36.49 ± 0.014 ^b^	70.17 ^b^	87.98 ^ab^	45.18 ± 0.23 ^c^	64.17 ± 1.47 ^b^
NOVS	35.95 ± 0.009 ^b^	70.61 ^b^	88.76 ^a^	49.16 ± 0.54 ^c^	73.43 ± 2.01 ^a^
P	40.74 ± 0.023 ^a^	83.12 ^a^	92.55 ^a^	146.39 ± 0.85 ^a^	61.28 ± 0.98 ^b^
HYT-F	41.44 ± 0.056 ^a^	79.62 ^ab^	77.96 ^b^	140.54 ± 0.98 ^a^	71.16 ± 1.94 ^a^
HYT-L	36.34 ± 0.021 ^b^	82.11 ^a^	88.95 ^a^	147.46 ± 1.36 ^a^	64.96 ± 2.88 ^b^

GAE: Gallic acid equivalents; SD: Standard deviation; Superscript letters indicate significant differences (*p* < 0.05) between natural extracts. P: Pomegranate extract, RA: Rosemary extract rich in Rosmarinic Acid; NOS: Rosemary extract rich in diterpenes; NOVS: Rosemary extract rich in diterpenes and with lecitin as emulsifier; HYT-L: Hydroxytyrosol extract obtained from olive leaf; HYT-F: Hydroxytyrosol extract obtained from olive fruit.

**Table 3:** Antimicrobial activity of natural extracts measured by the disc difussion method (mm ± SD).

Samples	Concentration (ppm)	Dilution (µL)	L. monocytogenes KCTC 3569 CECT 7467 (Gram-positive)	S. Aureus ATCC 25923 CECT 435 (Gram-positive)	E. Coli O157:H7 ATCC 25922 CECT 434 (Gram-negative)
RA	1000	30	8.0 ± 0.0	7.8 ± 0.7	6.9 ± 1.0
		60	9.6 ± 0.6	10.5 ± 0.5	12.3 ± 0.6
		90	12.3 ± 0.6	16.8 ± 1.8	15.2 ± 0.3
NOS	1000	30	8.0 ± 0.5	7.5 ± 0.0	8.0 ± 0.5
		60	10.8 ± 0.7	9.0 ± 0.5	10.5 ± 1.0
		90	14.0 ± 0.7	13.1 ± 0.5	20.0 ± 1.0
NOVS	1000	30	-	-	-
		60	7.7 ± 1.0	6.7 ± 0.5	7.2 ± 0.7
		90	14.7 ± 1.0	11.9 ± 0.3	18.5 ± 1.0
P	1000	30	10.3 ± 0.6	9.0 ± 0.0	8.0 ± 0.0
		60	12.5 ± 0.5	11.2 ± 0.3	10.2 ± 0.3
		90	15.3 ± 0.6	13.8 ± 0.8	12.6 ± 0.6
HYT-F	1000	30	6.0 ± 0.6	7.8 ± 0.5	-
		60	10.0 ± 0.6	14.5 ± 0.3	-
		90	13.0 ± 1.5	25.2 ± 0.5	11.3 ± 0.8
HYT-L	1000	30	10.0 ± 0.5	9.0 ± 0.5	-
		60	12.5 ± 0.6	18.5 ± 0.6	8.0 ± 0.5
		90	15.0 ± 0.5	28.1 ± 0.5	12.0 ± 0.5
Chloramphenicol	30 µM		34.7 ± 0.6	32.7 ± 0.5	33.7 ± 0.7

Chloramphenicol: positive control; SD: Standard Deviation. P: Pomegranate extract, RA: Rosemary extract rich in Rosmarinic Acid; NOS: Rosemary extract rich in diterpenes; NOVS: Rosemary extract rich in diterpenes and with lecitin as emulsifier; HYT-L: Hydroxytyrosol extract obtained from olive leaf; HYT-F: Hydroxytyrosol extract obtained from olive fruit.

**Table 4:** Average values and standard deviations of organic compounds (mg/g) in fish patties stored for 11 days, under retail conditions.

	Sample	Day 0 (M ± SD)	Day 11 (M ± SD)
1-Penten-3-ol	Control	0.05 ± 0.01	5.14 ± 0.01*^a^*
	P	0.06 ± 0.05	3.86 ± 0.05*^b^*
	RA	0.04 ± 0.02	3.54 ± 0.02*^b^*
	NOS	0.05 ± 0.04	1.95 ± 0.04*^c^*
	NOVS	0.01 ± 0.03	2.01 ± 0.03*^c^*
	HYT-F	0.04 ± 0.05	4.24 ± 0.05*^b^*
	HYT-L	0.07 ± 0.04	4.27 ± 0.04*^b^*
Hexanal	Control	0.03 ± 0.00	4.25 ± 0.02*^a^*
	P	0.09 ± 0.01	2.01 ± 0.03*^b^*
	RA	0.04 ± 0.02	2.96 ± 0.04*^b^*
	NOS	0.07 ± 0.01	1.53 ± 0.02*^b^*
	NOVS	0.02 ± 0.05	1.47 ± 0.02*^b^*
	HYT-F	0.03 ± 0.01	2.88 ± 0.05*^b^*
	HYT-L	0.03 ± 0.01	2.96 ± 0.03*^b^*
2-nonanone	Control	0.16 ± 0.01	0.39 ± 0.02
	P	0.51 ± 0.01	0.46 ± 0.04
	RA	0.37 ± 0.01	0.22 ± 0.03
	NOS	0.33 ± 0.01	0.16 ± 0.01
	NOVS	0.29 ± 0.01	0.25 ± 0.03
	HYT-F	0.47 ± 0.01	0.27 ± 0.05
	HYT-L	0.39 ± 0.01	0.32 ± 0.01
1,6-octadien-3-ol	Control	16.79 ± 0.04*^a^*	22.32 ± 0.21 *^a^*
	P	2.05 ± 0.04*^b^*	2.32 ± 0.05*^b^*
	RA	2.05 ± 0.03*^b^*	2.55 ± 0.03*^b^*
	NOS	0.65 ± 0.01*^b^*	0.44 ± 0.01*^b^*
	NOVS	0.77 ± 0.02*^b^*	0.77 ± 0.02*^b^*
	HYT-F	0.57 ± 0.02*^b^*	0.57 ± 0.02*^b^*
	HYT-L	0.87 ± 0.02*^b^*	0.87 ± 0.02*^b^*
Nonanal	Control	0.16 ± 0.01	1.81 ± 0.02
	P	0.51 ± 0.01	1.30 ± 0.02
	RA	0.37 ± 0.01	1.75 ± 0.02
	NOS	0.33 ± 0.01	0.66 ± 0.02
	NOVS	0.29 ± 0.01	0.66 ± 0.01
	HYT-F	0.47 ± 0.01	1.20 ± 0.03
	HYT-L	0.39 ± 0.01	1.57 ± 0.02
Pentadecane	Control	1.13 ± 0.02	1.60 ± 0.02
	P	1.17 ± 0.03	1.44 ± 0.02
	RA	0.10 ± 0.0	1.04 ± 0.03
	NOS	0.62 ± 0.01	1.08 ± 0.01
	NOVS	0.57 ± 0.01	1.05 ± 0.03
	HYT-F	0.82 ± 0.03	1.54 ± 0.01
	HYT-L	0.75 ± 0.03	1.27 ± 0.01

Results are expressed as mean ± standard deviation in arbitrary area units (× 10^6^). P: Pomegranate extract, RA: Rosemary extract rich in Rosmarinic Acid; NOS: Rosemary extract rich in diterpenes; NOVS: Rosemary extract rich in diterpenes and with lecitin as emulsifier; HYT-L: Hydroxytyrosol extract obtained from olive leaf; HYT-F: Hydroxytyrosol extract obtained from olive fruit.

**Table 5:** Microbiological results (cfu/g) of fish patties analysis at days 0, 4, 7 and 11 under refrigerated storage.

Analysis	Sample	Storage day			
		0	4	7	11
TVC	Control	1.98 × 10^3^	6.20 × 10^3^	2.77 × 10^4^	5.55 × 10^7^
	P	1.52 × 10^3^	5.12 × 10^3^	1.28 × 10^4^	5.35 × 10^7^
	RA	9.10 × 10^2^	4.25 × 10^3^	2.01 × 10^4^	7.29 × 10^7^
	NOS	7.25 × 10^2^	3.62 × 10^3^	1.10 × 10^4^	6.50 × 10^7^
	NOVS	1.01 × 10^3^	4.05 × 10^3^	1.56 × 10^4^	6.90 × 10^7^
	HYT-L	1.88 × 10^3^	6.22 × 10^3^	1.79 × 10^4^	3.48 × 10^7^
	HYT-F	2.01 × 10^3^	5.98 × 10^3^	2.10 × 10^4^	3.15 × 10^7^
TCC	Control	<10	8.40 × 10^2^	4.75 × 10^3^	5.45 × 10^4^
	P	<10	1.40 × 10^3^	7.80 × 10^3^	6.75 × 10^3^
	RA	<10	1.95 × 10^3^	6.10 × 10^3^	3.78 × 10^4^
	NOS	<10	1.32 × 10^3^	5.30 × 10^3^	2.63 × 10^4^
	NOVS	<10	1.69 × 10^3^	6.00 × 10^3^	3.12 × 10^4^
	HYT-L	<10	4.4 × 10^2^	3.30 × 10^3^	7.15 × 10^4^
	HYT-F	<10	5.9 × 10^2^	3.90 × 10^3^	7.81 × 10^4^
*E. Coli*	Control	<10	<10	<10	<10
	P	10	<10	<10	<10
	RA	<10	<10	<10	<10
	NOS	<10	<10	<10	<10
	NOVS	<10	<10	<10	<10
	HYT-L	<10	<10	20	<10
	HYT-F	<10	<10	<10	<10
*L. monocytogenes*	Control	Absence in 25 g	Absence in 25 g	Absence in 25 g	Absence in 25 g
	P	Absence in 25 g	Absence in 25 g	Absence in 25 g	Absence in 25 g
	RA	Absence in 25 g	Absence in 25 g	Absence in 25 g	Absence in 25 g
	NOS	Absence in 25 g	Absence in 25 g	Absence in 25 g	Absence in 25 g
	NOVS	Absence in 25 g	Absence in 25 g	Absence in 25 g	Absence in 25 g
	HYT-L	Absence in 25 g	Absence in 25 g	Absence in 25 g	Absence in 25 g
	HYT-F	Absence in 25 g	Absence in 25 g	Absence in 25 g	Absence in 25 g

TVC: Total Viable Count; TCC: Total Coliform Count P: Pomegranate extract, RA: Rosemary extract rich in Rosmarinic Acid; NOS: Rosemary extract rich in diterpenes; NOVS: Rosemary extract rich in diterpenes and with lecitin as emulsifier; HYT-L: Hydroxytyrosol extract obtained from olive leaf; HYT-F: Hydroxytyrosol extract obtained from olive fruit.
